# Retraction: Activation of the IL-1β/KLF2/HSPH1 pathway promotes STAT3 phosphorylation in alveolar macrophages during LPS-induced acute lung injury

**DOI:** 10.1042/BSR20193572_RET

**Published:** 2026-09-22

**Authors:** Yafeng Liang, Jiaqi Luo, Nengli Yang, Shufen Wang, Mingwei Ye, Guoquan Pan

**Affiliations:** 1 Department of Pediatric Intensive Care Unit, The Second Affiliated Hospital and Yuying Children's Hospital, Wenzhou Medical University, Wenzhou 325000, China; 2 Department of Anesthesiology, The First Affiliated Hospital of Wenzhou Medical University, Wenzhou 325000, Zhejiang, China; 3 Department of Pediatrics, Sanmen People's Hospital of Zhejiang, Sanmen 317100, China

**Keywords:** acute lung injury, alveolar macrophages, heat shock protein H1, interleukin-1β, kruppel like factor 2, p-STAT3

This article is being retracted from *Bioscience Reports* at the request of the Editor-in-Chief and the Editorial Board. This follows the identification of concerns involving Figures 2, 3, 5 and 6 of the article, alerting the Editorial Board to similarities between the paper and these published articles: https://doi.org/10.1186/s11658-018-0117-x and https://doi.org/10.1042/BSR20180532.

Specifically, these similarities include:

1. The Figure 2D GADPH Western blot appears highly similar to that of Figure 4C GADPH Western blot of Liang et al. (2018). DOI: 10.1186/s11658-018-0117-x
2. The Figure 2C GADPH Western blot appears highly similar to that of Figure 6F GADPH Western blot of Gong et al. (2018). DOI: 10.1042/BSR20180532
3. The Figure 3E p-STAT3 Western blot appears highly similar, when flipped horizontally, to that of Figure 6F MMP14 Western blot of Gong et al. (2018). DOI: 10.1042/BSR20180532
4. The Figure 5C GADPH Western blot appears highly similar to that of Figure 2G GADPH Western blot of Gong et al. (2018). DOI: 10.1042/BSR20180532
5. Figure 2D STAT3 Western blot appears highly similar to that of Figure 3E STAT3 Western blot
6. Figure 2C GADPH Western blot appears highly similar to that of Figure 3B GADPH Western blot
7. Figure 3B GADPH Western blot appears highly similar, when flipped horizontally, to that of Figure 3C GADPH Western blot
8. Figure 3E p-STAT3 Western blot appears highly similar, when flipped horizontally, re-scaled and darkened, to that of Figure 6F MMP14 Western blot
9. Figure 2D STAT3 Western blot appears highly similar to that of Figure 3E STAT3 Western blot
10. Figure 6G p-STAT3/LPS + KNK437 histology image appears highly similar to that of Figure 6G STAT3/NC histology image

The authors have been contacted with regards to the retraction and have provided a response which disclosed that a third party biotechnology company was used for all Western Blot experiments and their data had been mixed up with that of another project, hence the similarities with other papers. As we require authors to disclose any third parties during submission and the authors have not respond to our further queries on this, we have lost confidence in the data presented within the paper. Furthermore, the original Western Blot images provided within the author response did not meet the standard of the Journal and raised further discrepancies between the raw blot for Figure 3 and the Figure 3E STAT3, with background detail appearing different. This was raised to the authors and requested that they provide an explanation for any discrepancies, however we have received no response.

Given the extent of the issues raised, the Editorial Board stands by the decision to retract the article.

